# Perinatal interventions for parents with exposure to adverse childhood experiences: A narrative review of suitability for implementation in primary care

**DOI:** 10.1002/imhj.70095

**Published:** 2026-05-08

**Authors:** Joann J. Chen, Katherine Magnuson, Veronica Bordes Edgar, Sunita M. Stewart

**Affiliations:** ^1^ The University of Texas Southwestern Medical Center Dallas Texas USA; ^2^ Children's Medical Center Dallas Texas USA

**Keywords:** Adverse childhood experiences, implementation, intervention, perinatal, primary care

## Abstract

Children of parents exposed to adverse childhood experiences (ACEs) face elevated risk of experiencing biopsychosocial challenges. Primary care screening for parental ACEs is increasing, and routine primary care touchpoints may reach parents with exposure to ACEs during the critical perinatal period. This review seeks to 1) evaluate existing perinatal interventions—including those not designed for primary care—for suitability for primary care implementation and 2) identify implementation factors missing from existing interventions that may enhance their implementation in primary care. The PubMed database was searched for articles in English reporting on randomized controlled trials of perinatal interventions among parents with exposure to ACEs. Fourteen articles detailing 14 unique interventions were included in the qualitative synthesis. Data were extracted on intervention characteristics identified by implementation research as relevant to primary care: evidence of benefit, applicability and relevance, complexity, clarity, practicality and utility, costs, and adaptability. Ten interventions were found to be effective for parents with greater exposure to ACEs; however, the variability within the interventions and relevant settings suggests nuanced decision‐making is required to match interventions to settings. The current review offers primary care providers a framework for matching and adapting such interventions.

## 1 INTRODUCTION

Childhood exposure to abuse, neglect, and other adverse interpersonal experiences can have lasting impact on individuals and on society. A growing body of interventions for parents seeks to disrupt biopsychosocial mechanisms in the perinatal period that are relevant to the intergenerational impact of such experiences. Despite frequent primary care engagement in the perinatal period, increased screening for adverse childhood experiences (ACEs) in primary care, and the alignment of the function of primary care with these interventions, few such interventions have been evaluated for suitability for implementation in primary care settings or have been implemented in such settings. Various factors contribute to the gap in implementation of these interventions in primary care, including characteristics of the interventions themselves. The current review seeks to support primary care clinicians and researchers in narrowing the gap between the evidence for these interventions broadly and their implementation in primary care specifically by detailing intervention characteristics that existing literature has found to promote intervention implementation within primary care. We additionally offer an evaluation framework for assessing the suitability of interventions for a given primary care setting. Acknowledging the diversity within primary care settings, within ACEs, and within existing perinatal interventions for parents with exposure to ACEs, the current study seeks not to compare existing interventions and identify an “optimal” intervention but, instead, to offer insight for primary care decision‐makers to match and adapt interventions to their unique settings and needs.

### 1.1 ACEs and the intergenerational transfer of adverse outcomes

ACEs consist of potentially traumatic events that occur before the age of 18. Many types of events have been considered ACEs (SmithBattle et al., 2022). The original ACEs study included ten such events that continue to be commonly included in the definition of ACEs: physical, emotional, and sexual abuse; physical and emotional neglect; parental separation or divorce; and household dysfunction, including violence, substance use, mental illness or suicide attempt, and incarceration among household members (Felitti et al., 1998; Zarse et al., 2019). In a sample of U.S. adults, 25% reported having experienced three or more ACEs (Merrick et al., 2018), and 44% of mothers reported having experienced childhood abuse or neglect (Moog et al., 2023). Maternal exposure to ACEs is associated with children's internalizing and externalizing symptoms (Plant et al., 2018), psychological and medical diagnoses (Moog et al., 2023), and subsequent exposure to trauma (Lange et al., 2019). Paternal exposure to ACEs has also been associated with more trauma symptoms in both fathers and their children (Lünnemann et al., 2019). Throughout the current study, we specify when findings relate to mothers, fathers, parents, or caregivers in general. We use the term parents as a general term for caregivers when not referring to a specific study's findings.

**Key Findings**

- Several existing perinatal interventions seeking to improve parent, parenting, and infant outcomes are effective for parents with exposure to ACEs.
- Few effective perinatal interventions for parents with exposure to ACEs have been designed for, evaluated in, or implemented in primary care, despite increasing rates of ACEs screening in primary care.
- Variability across ACEs, across interventions, and across primary care settings requires clinicians to match and adapt interventions to their specific primary care settings.

**Statement of Relevance**

The narrative review fills a gap in understanding how existing treatments can be leveraged to interrupt potential intergenerational transfer of adverse outcomes at the earliest stages of child development. By mapping existing interventions to key implementation characteristics identified as critical to primary care implementation, the review helps primary care decision‐makers identify interventions that are relevant to their unique perinatal settings. The findings further encourage perinatal primary care providers to consider opportunities to engage with parents with exposure to ACEs from pregnancy through infancy, thereby offering preventative support to infants and families to enhance mental and physical health outcomes.

### 1.2 Perinatal mediators of trauma transfer

Key mediators of the intergenerational transfer of adverse outcomes following parental exposure to ACEs suggest potential for intervening during the perinatal period (Chamberlain et al., 2019), commonly defined as encompassing pregnancy through the first two years of life. Recognized perinatal mediators of the association between maternal exposure to ACEs and child mental, physical, and social outcomes include hypothalamic‐pituitary‐adrenal (HPA) axis dysregulation in pregnancy, higher rates of perinatal depression, higher prevalence of breastfeeding complications, higher rates of insecure attachment, and lower parenting confidence (Elfgen et al., 2017; Ishikawa et al., 2022; Julian et al., 2022; Lotto et al., 2023; Plant et al., 2018; Smith et al., 2016). These findings highlight the potential for intervening in the perinatal period, a time when parental behavior may be particularly receptive to change (Fava et al., 2016; Massey et al., 2017).

Aligned with growing interest in perinatal intervention for parents with exposure to ACEs, Jones and colleagues (2023) conducted a systematic review of perinatal interventions for parents with child maltreatment histories or complex posttraumatic stress disorder (CPTSD) symptoms. The review found that although additional high‐quality evidence is needed, current literature suggests that certain perinatal parenting and psychological interventions may improve outcomes for parents with childhood maltreatment histories or symptoms of CPTSD (Jones et al., 2023).

### 1.3 The potential of primary care

For perinatal interventions found to be effective for parents with a history of childhood maltreatment and other ACEs, there is unexplored potential for implementation within primary care, defined as integrated healthcare services provided by clinicians who administer most of a patient's healthcare needs through sustained partnership (Oddone & Boulware, 2016). Primary care providers include those within family medicine, internal medicine, and pediatrics and often also include those within obstetrics, who offer comprehensive and preventative care for many women of childbearing age (Nettleman & Yanni, 2003).

The primary care setting may be uniquely suited for implementation of perinatal intervention for parents with ACEs. Such interventions can leverage frequent primary care touchpoints as current guidance recommends 24 obstetric and pediatric visits from pregnancy through the second year of life (Kilpatrick, 2017). Primary care interventions may be able to reach most families, as 98.4% of pregnant individuals in the U.S. receive some prenatal care and well child visit adherence is estimated at 62.3% (Abdus & Selden, 2022; Osterman & Martin, 2018). Furthermore, integrating interventions into routine care settings may reduce stigma and increase uptake (Buchanan et al., 2023).

Beyond the logistical advantages of primary care for perinatal intervention, the core functions of primary care—first contact, comprehensiveness, coordination, and continuity—may offer unique opportunities to address the increased risks related to parental exposure to ACEs (Jimenez et al., 2021). As patients’ first point of contact within the healthcare system, primary care providers offer accessible and generalist support, which can include screening for exposure to ACEs. Primary care's role of offering comprehensive rather than specialized care can also include evaluation of the impact of parental ACEs across biopsychosocial domains, leveraging multidisciplinary staff. Following comprehensive assessment, primary care teams can coordinate care with appropriate specialists across relevant domains potentially impacted by parental ACEs. Finally, primary care teams offer longer‐term patient‐provider relationships than are typically available with specialists, allowing for continuity of care.

### 1.4 ACEs screening in primary care

The opportunity within primary care for identifying and addressing ACEs is reflected in the increase in ACEs screening for adults, children, and caregivers in primary care settings. The American Academy of Pediatrics (AAP), while stopping short of recommending ACEs screening specifically, recommends using standardized measures to identify families’ risk factors for toxic stress, which include ACEs (Campbell, 2020; Garner & Yogman, 2021). Clinicians and researchers continue to debate the utility of universal ACEs screening in primary care settings (Bair‐Merritt & Zuckerman, 2016; Gupta et al., 2021). For critics of widespread screening, objections include concerns regarding acceptability of screening among patients and providers, lack of standardized screening tools, and lack of evidence for ACEs questionnaires as clinical screening instruments versus population‐level research tools (Austin et al., 2024; SmithBattle et al., 2022). Despite the ongoing debate, ACEs screening in primary care settings is expanding. In California alone, the ACEs Aware initiative launched in 2020 has resulted in over 3.6 million ACEs screenings as of March 2024, including 16.5% of Medicaid beneficiaries (RAND Corporation, 2025).

The increase in ACEs screening may, however, be outpacing the development of guidelines for appropriate guidance and intervention following positive screenings, creating a challenge for primary care providers. In a study of California primary care providers, one of the top barriers to implementing ACEs screening was the “unclear treatment pathway for detected ACEs” (Viglione et al., 2025). Critics of parental ACEs screening in pediatric primary care have similarly highlighted the need for evidence to support a recommended response to positive screening (Bair‐Merritt & Zuckerman, 2016). Limitations of many ACEs screening tools may contribute to clinicians’ challenges in treatment planning following positive ACEs screenings. Many instruments that screen for ACEs assess only the number of types of adverse experiences within a specific set of potentially traumatic experiences, which may include experiences as different as bullying by a peer and physical neglect by a caregiver (Holden et al., 2020). The breadth of ACEs is clinically relevant because outcomes differ by ACE; even within childhood abuse and neglect, different forms of abuse or neglect have been associated with different outcomes (Strathearn et al., 2020). Furthermore, commonly used ACEs screening instruments may not evaluate the frequency, duration, and impact of those experiences, which limits their utility in treatment planning because exposure to an ACE is not necessarily traumatic (Holden et al., 2020).

### 1.5 Connecting evidence and practice in primary care

A gap between evidence and practice is thus evident when considering that 1) early evidence from Jones and colleagues (2023) shows some interventions are effective for parents with exposure to ACEs and 2) clinicians are not aware of appropriate interventions for parents exposed to ACEs. This so‐called evidence‐practice gap has been found across settings and results in 30%–40% of patients not receiving care in line with current scientific evidence (Schuster et al., 1998).

Researchers seeking to reduce the evidence‐practice gap have identified intervention characteristics that promote implementation (Huybrechts et al., 2021; Lau et al., 2015). Lau et al. (2015) conducted a systematic review of reviews to identify causes of the evidence‐practice gap for complex interventions, including mental health interventions, in primary care settings. They further sought to elucidate the factors that affect successful implementation of interventions within those settings. The review identified factors related to a primary care setting's external context, organization, and professionals that impact implementation. It also identified nine factors related to the “nature and characteristics” of interventions that limit or facilitate their implementation in primary care:

1. Evidence of benefit—support for an intervention's efficacy or effectiveness relative to a control,
2. Applicability and relevance—evidence within relevant scope and population,
3. Complexity—lack of confusing and complex procedures or recommendations,
4. Clarity—lack of uncertainty regarding application of the intervention,
5. Practicality and utility—ease of use of the intervention,
6. Costs—low expenditure of resources,
7. adaptability—ability to modify the intervention to local circumstances,
8. Cost‐effectiveness—low cost of an intervention relative to alternative options, and
9. IT compatibility—interoperability with existing systems.

In the current study, we use these factors identified by Lau and colleagues but exclude IT compatibility and cost‐effectiveness because these factors focus on characteristics that vary by primary care setting and fall outside the scope of the current review. Acknowledging the continuum from efficacy (i.e., evaluating under ideal conditions) to effectiveness (i.e., evaluating under “real world” conditions) within evidence of benefit, we refer to intervention effectiveness due to our focus on primary care implementation and the included studies’ integration of key elements of effectiveness trials (e.g., clinically relevant treatment modalities) (Gartlehner et al., 2006).

### 1.6 The current study

The current narrative review has two overlapping aims: 1) for primary care providers and administrators who are screening for or considering screening for parental ACEs, we evaluate existing perinatal interventions for suitability for implementation specifically in primary care settings and 2) for researchers developing and evaluating perinatal interventions for parents with exposure to ACEs, we identify implementation factors missing from the existing landscape of interventions that may enhance their potential implementation in primary care settings. This study seeks to clarify the strengths and gaps in existing intervention literature in meeting the needs of parents exposed to ACEs through primary care.

## 2 Methods

### 2.1 Search strategy

We conducted searches of PubMed in February 2024 for articles with the following terms in the title or abstract: (((random*) AND (trauma OR ACE OR adverse childhood experience* OR adverse childhood event* OR maltreatment OR abuse OR neglect)) AND (intervention* OR program* OR *therapy OR *therap*)) AND (*natal* OR fetal OR mother OR father OR parent* OR maternal OR paternal OR caregiver).

### 2.2 Inclusion criteria

For inclusion, articles were required to meet all five inclusion criteria:

1. Had full text versions available in English,
2. Were published in January 2024 or before,
3. Reported on results of a randomized controlled trial to ensure rigorous evidence of benefit,
4. Reported outcomes of an intervention delivered during the perinatal period, and
5. Reported such outcomes specifically for parents with exposure to ACEs.

Studies that included participants older than two years of age were included if the mean age was under two years. We excluded review articles but perused them for relevant citations. We included all definitions of ACEs that included childhood maltreatment or interpersonal trauma.

### 2.3 Study selection and data extraction

**FIGURE 1:**
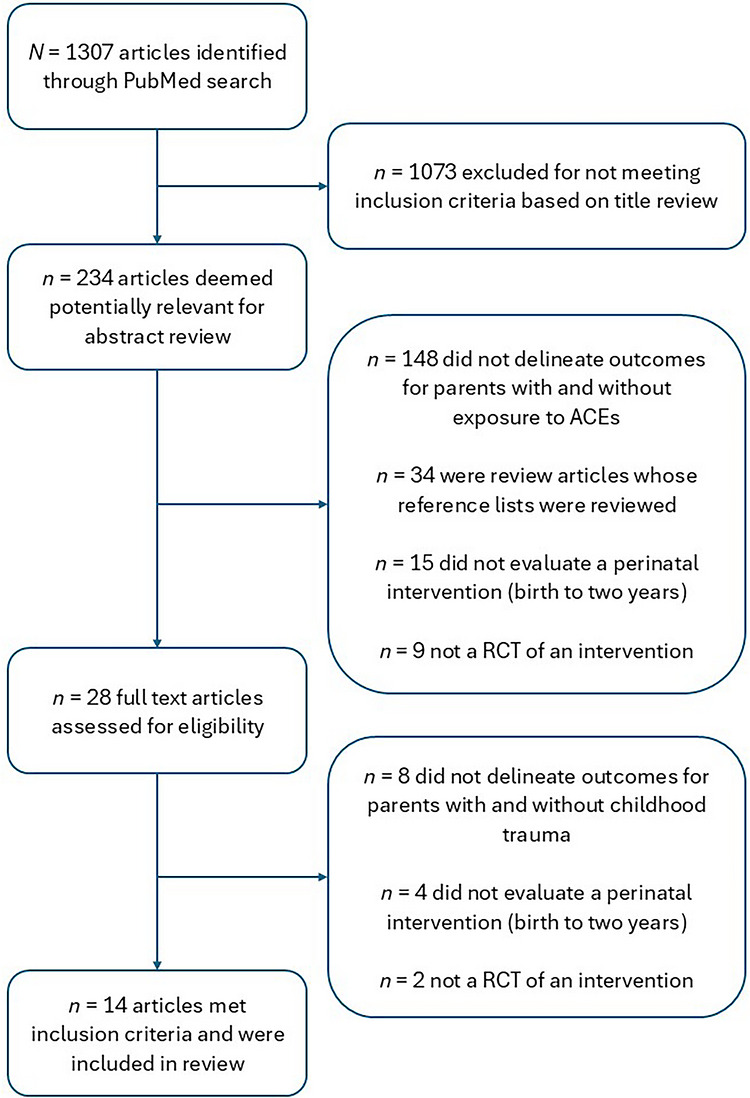
Flowchart of study selection. RCT, randomized controlled trial.

Titles and abstracts from the database searches were reviewed for relevance. Those selected for further review were read in full, and inclusion decisions were made based on the full content (Figure 1). Key article information and study characteristics were extracted.

Study findings on treatment outcomes for parents with relatively higher exposure to ACEs (r+ACE) were extracted. Studies differed in their definitions of ACEs and comparisons of ACEs exposure; therefore, the definition of r+ACE varies by study, the implications of which are discussed below. R+ACE effective interventions were defined as those interventions that, for at least one outcome, had significant response among r+ACE participants that was either equal to or more favorable than the response for participants with less exposure to ACEs.

Additional data extracted aligned with the relevant implementation concepts of evidence of benefit, applicability and relevance, complexity, clarity, practicality and utility, costs, and adaptability. The current review consists of summary and qualitative synthesis of the results of this data extraction.

## 3 RESULTS

Fourteen studies met the inclusion criteria, reporting on 14 different interventions (Ammerman et al., 2016; Berry et al., 2021; Blalock et al., 2013; Goldstein et al., 2024; Grote et al., 2012; Liu et al., 2021; Pasalich et al., 2019; Perrone et al., 2021; Ribaudo et al., 2022; Riem et al., 2021; Rosenblum et al., 2017; Steele et al., 2019; Van Der Asdonk et al., 2021). For ease of reference, we refer to each study by the study number from Table 1, which summarizes key study characteristics assessed. For three studies (1, 3, 6), some relevant data were not reported in the included articles and were thus extracted from related articles (Ammerman et al., 2013; Cinciripini et al., 2010; Grote et al., 2009). Only one study (12) specifically cited an implementation‐related goal within the study aims.

**TABLE 1:** Summary characteristics of included studies.

Study #	Study (first author, year)	Intervention	Intervention description	Program timing	Participants	Sample	Designed for ACEs history?	ACEs exposure assessment	Exposure to ACEs comparison	Outcomes for which intervention was effective for r+ACEs parents
(1)	Ammerman et al., 2016, 2013 Ammerman et al., 2013a; Ammerman et al., 2013b	In‐Home Cognitive Behavioral Therapy (IH‐CBT)	Cognitive behavioral therapy concurrent with home visiting	Pregnancy through postpartum	93 mothers	Clinical: MDD diagnosis	No	CTQ‐SF	Used continuous variable of ACEs exposure	Decrease in maternal depressive symptoms
(2)	Berry et al., 2021	Practical Resources for Effective Postpartum Parenting (PREPP)	Newborn skills coaching, psychological support, and psychoeducation aligned with prenatal visits	Pregnancy through postpartum	109 mother‐child dyads	Community	No	CTQ‐SF	Compared mothers with at least one type of child maltreatment to those with none	Increase in infant daytime sleep
(3)	Blalock et al., 2013 & Cinciripini et al., 2010	Cognitive Behavioral Analysis System of Psychotherapy (CBASP)	Integrative psychotherapy focused on improving relationships	Pregnancy	248 pregnant women	Clinical: Tobacco use	No	CTQ‐SF	Used continuous variable of ACEs exposure	Decrease in maternal depressive symptoms
(4)	Condon et al., 2022	Minding the Baby (MtB)	Home visiting intervention for first time mothers with range of strategies (e.g., health education, parenting skills education)	Pregnancy through postpartum	97 mother‐child dyads	Community	No	CTQ‐SF	Used continuous variable of ACEs exposure	Decrease in maternal hostile/coercive parenting
(5)	Goldstein et al., 2024	Mothers and Babies Personalized (MB‐P)	Cognitive behavioral intervention to encourage positive activities and thought patterns and strengthen parental bond	Pregnancy	95 pregnant women	Community	No	ACEs Questionnaire—modified	Compared women with at least one ACE to those with none	–
(6)	Grote et al., 2012 & Grote et al., 2009	Culturally Relevant Brief Interpersonal Psychotherapy (IPT‐B)	Interpersonal therapy designed for treatment engagement and addressing barriers to care	Pregnancy	53 pregnant women	Clinical: Depression	No	CTQ‐SF	Used continuous variable of ACEs exposure	–
(7)	Liu et al., 2021	Filming Interactions to Nurture Development (FIND)	Strengths‐based video feedback intervention to strengthen parent‐child interactions	Postpartum	91 caregiver‐child dyads; 98.9% mothers	Community	No	Unvalidated ACEs measure	Used continuous variable of ACEs exposure	Increase in parental sense of competence; increase in parental self‐efficacy in teaching
(8)	Pasalich et al., 2019	Promoting First Relationships (PFR)	Strengths‐based consultation focused on increasing parental sensitivity with video feedback	Postpartum	247 parent‐child dyads; > 90% mothers	Other: Open case of maltreatment	No	CTQ‐SF	Compared parents with moderate to severe abuse by category to those with no or mild abuse by category	Increase in parental sensitivity; increase in child secure base behavior
(9)	Perrone et al., 2021	Attachment and Biobehavioral Catch‐up (ABC)	Home visiting program to enhance nurturance and responsiveness and reduce frightening behaviors with in‐the‐moment feedback	Postpartum	200 caregiver‐child dyads; 96% mothers	Community	No	ACEs Questionnaire	Used continuous variable of ACEs exposure	–
(10)	Ribaudo et al., 2022	Michigan Model of Infant Mental Health‐Home Visiting (IMH‐HV)	Needs‐based home visiting intervention with range of strategies (e.g., emotional support, infant‐child psychotherapy)	Postpartum	58 mother‐child dyads	Community: High risk	No	ACEs Questionnaire	Used continuous variable of ACEs exposure	–
(11)	Riem et al., 2021	Baby carrier intervention (BCI)	Instruction on and encouragement to use ergonomic soft baby carrier	Postpartum	63 first‐time fathers	Community	No	CTS	Used continuous variable of ACEs exposure	Increase in paternal amygdala reactivity to infant crying
(12)	Rosenblum et al., 2017	Mom Power	Multifamily, group‐based intervention with tailored feedback to enhance understanding of children's behaviors	Postpartum	122 mother‐child dyads	Community: High risk	Designed for mothers with any trauma or abuse history	LSC—modified	Compared mothers with at least one exposure to interpersonal trauma to those with none	Decrease in maternal depressive symptoms; decrease in maternal PTSD symptoms
(13)	Steele et al., 2019	Group Attachment‐Based Intervention (GABI)	Multifamily, group‐based intervention with video feedback and attention to parent, child, and parent‐child relationship	Postpartum	78 mother‐child dyads; paternal engage‐ment not reported	Community: High risk	Designed for mothers with trauma history or other risk factors for child maltreatment	Clinical ACEs Questionnaire	Compared mothers with at least four ACEs to those with less than four ACEs	Increase in maternal supportive presence; decrease in maternal hostility
(14)	van der Asdonk et al., 2021	Attachment Video‐feedback Intervention (AVI)	Short‐term video feedback intervention enhancing sensitive parenting behaviors and reducing frightening behaviors	Postpartum	88 caregiver‐child dyads; 86% mothers	Other: Substantiated child maltreatment	No	CTQ	Used continuous variable of ACEs exposure	–

Abbreviations: ACE, adverse childhood experience; CTQ, Childhood Trauma Questionnaire; CTQ‐SF, Childhood Trauma Questionnaire—Short Form; CTS, Conflict Tactics Scale—Parent Child; LSC, Life Stressor Checklist; MDD, major depressive disorder; r+ACE, relatively higher exposure to ACEs.

Extracted data related to implementation characteristics are listed in Table 2. Some data related to multiple implementation characteristics; we included each variable only once to avoid redundancy. Intervention evidence of benefit data are summarized in Table 3 and detailed in Table S1. Other intervention implementation characteristics are detailed in Table S2.

**TABLE 2:** Extracted intervention characteristics.

	Evidence of benefit	Applicability and relevance	Complexity	Clarity	Practicality and utility	Costs	Adaptability
Defined primary and secondary intervention outcomes	✓						
Intervention effectiveness against defined outcomes: main treatment effect among relatively higher ACEs parents, relatively lower ACEs parents, and full sample; interaction effect of treatment and ACEs status; intervention efficacy for r+ACE parents	✓						
Type of control group	✓						
Description of control group	✓						
Length of follow‐up	✓						
Designed for parents with ACEs history: yes/no		✓					
Program timing: prenatal, postnatal or both		✓					
Completion rate		✓					
Definition of completion		✓					
Utilization rate(s) and/or other acceptability measures		✓					
Sample size and participant description	✓	✓					✓
Type of sample: community, clinical, other		✓					✓
ACEs prevalence in sample		✓					✓
Demographics of sample, incl. race/ethnicity and income level		✓					✓
Treatment standardization (e.g., use of manuals)			✓	✓	✓		
Education/certification level of professional delivering intervention			✓		✓	✓	
Training required to administer intervention			✓	✓	✓	✓	
Number of intervention sessions					✓	✓	
Session length					✓	✓	
Total intervention hours					✓	✓	
Intervention mode and setting: individual vs. group, in‐person versus virtual, in home versus clinic versus community			✓		✓	✓	
Reported intervention adaptability to individual or to population		✓					✓

Abbreviations: ACE, adverse childhood experience; r+ACE, relatively higher exposure to ACEs.

**TABLE 3:** Intervention effectiveness for r+ACE parents by outcome measure.

Outcome measure	Study #	Intervention	Intervention effectiveness for r+ACE parents	Reported effect size
Parental Outcomes				
Decrease in maternal depressive symptoms	(1)	IH‐CBT	Similar	Moderate
Decrease in maternal depressive symptoms	(3)	CBASP	Greater	(Not reported)
Decrease in maternal depressive symptoms	(6)	IPT‐B	Similar	Large
Decrease in maternal depressive symptoms	(12)	Mom Power	Not reported	Small to moderate
Decrease in maternal PTSD symptoms	(12)	Mom Power	Not reported	Small to moderate

Parenting Outcomes				
Decrease in maternal hostile/coercive parenting	(4)	MtB	Similar	(Not reported)
Increase in parental sense of competence	(7)	FIND	Similar	(Not reported)
Increase in parental self‐efficacy in teaching	(7)	FIND	Greater	(Not reported)
Increase in parental sensitivity	(8)	PFR	Greater, for physical abuse only	(Not reported)
Increase in paternal amygdala reactivity to infant crying	(11)	BCI	Greater	(Not reported)
Increase in maternal supportive presence	(13)	GABI	Similar	Small
Decrease in maternal hostility	(13)	GABI	Similar	Small

Child Outcomes				
Increase in infant daytime sleep	(2)	PREPP	Greater	(Not reported)
Increase in child secure base behavior	(8)	PFR	Greater, for physical abuse only	(Not reported)

Abbreviations: BCI, baby carrier intervention; CBASP, Cognitive Behavioral Analysis System of Psychotherapy; FIND, Filming Interactions to Nurture Development; GABI, Group Attachment‐Based Intervention; IH‐CBT, In‐Home Cognitive Behavioral Therapy; IPT‐B, Culturally Relevant Brief Interpersonal Psychotherapy; MtB, Minding the Baby; PFR, Promoting First Relationships; PREPP, Practical Resources for Effective Postpartum Parenting; PTSD, posttraumatic stress disorder; r+ACE, relatively higher exposure to adverse childhood experiences.

### 3.1 Evidence of benefit for r+ACE parents

Studies varied in their measures of ACEs, with the most common being the Childhood Trauma Questionnaire (CTQ) (*n* = 7; 1, 2, 3, 4, 6, 8, 14) and the original ACEs questionnaire or its variants (*n* = 5; 5, 7, 9, 10, 13) (Bernstein et al., 1994, 2003; Felitti et al., 1998). Most studies (*n* = 9; 1, 3, 4, 6, 7, 9, 10, 11, 14) compared higher and lower exposure to ACEs using a continuous variable; the remainder (2, 5, 8, 12, 13) set cutoffs to define two comparison groups.

Over 70% of interventions (*n* = 10; 1, 2, 3, 4, 6, 7, 8, 11, 12, 13) were found effective for r+ACE parents on at least one outcome. Four interventions (1, 3, 6, 12) were effective for parental outcomes (e.g., decrease in maternal depressive symptoms), five (4, 7, 8, 11, 13) for parenting outcomes (e.g., increase in parental self‐efficacy), and two (2, 8) for child outcomes (e.g., increase in child secure base behavior). Only four studies of r+ACE effective interventions (1, 6, 12, 13) directly reported effect sizes, which ranged from small (13) to large (6). In three studies (3, 13, 14), not enough information was reported to determine the treatment effect for certain outcomes among r+ACE parents.

### 3.2 Applicability and relevance

Two r+ACE effective interventions (12, 13) were designed for mothers with histories of trauma or abuse; none were designed specifically for parents with exposure to childhood trauma, childhood abuse, or other ACEs. Five of the r+ACE effective interventions (7, 8, 11, 12, 13) were delivered exclusively postnatally; only two were delivered exclusively prenatally (3, 6). Among r+ACE effective interventions, only one intervention (13) had a completion rate below 65%. High utilization was noted within three (3, 7, 8) of the four (3, 7, 8, 11) studies that reported utilization.

Seven studies of r+ACE effective interventions (1, 2, 3, 4, 6, 12, 13) included only mothers or mother‐child dyads, two included parents or caregivers of either gender (7, 8), and one included only fathers (11). The interventions targeting parental outcomes (1, 3, 6, 12) utilized clinical (1, 3, 6) or high‐risk community samples (12), while those targeting parenting outcomes (4, 7, 8, 11, 13) primarily utilized community samples (4, 7, 11). Prevalence of greater ACEs exposure ranged from 32% (2) to over 80% (1, 14). Seven r+ACE effective interventions (2, 3, 4, 6, 7, 12, 13) utilized samples primarily composed of racial or ethnic minority participants. In eight studies (1, 2, 3, 6, 7, 8, 11, 12), at least half of participants endorsed a measure related to low income.

### 3.3 Complexity and clarity

Only two studies of r+ACE effective interventions (1, 11) did not report using treatment standardization procedures. Six r+ACE effective interventions (1, 2, 3, 4, 6, 8) required at least a master's level clinician. Four of the r+ACE effective interventions (1, 2, 6, 11) did not report directly on training requirements for intervention providers.

### 3.4 Practicality, utility, and costs

Across r+ACE effective interventions, the number of sessions ranged from one (11) to over ninety sessions (4). Session length ranged from one (1, 3, 4, 8) to three hours (12); four studies (2, 6, 7, 11) did not report session length. Eight of the r+ACE effective interventions (1, 2, 3, 4, 6, 7, 8, 11) were delivered exclusively in individual settings. All interventions reviewed were delivered in person. Five r+ACE effective interventions were delivered exclusively in patients’ homes (1, 4, 7, 8, 11), three in clinics (2, 6, 13), and one across multiple settings (12). Of the three interventions delivered in clinic, two (2, 6) were delivered in primary care settings, specifically obstetrics and gynecology clinics.

### 3.5 Adaptability

Among r+ACE effective interventions, five (1, 2, 4, 12, 13) reported adaptability of the intervention to individual needs, and two (1, 6) reported intervention adaptability across populations.

## 4 DISCUSSION

The current review sought to evaluate existing perinatal interventions for parents with exposure to ACEs for suitability for implementation in primary care settings to 1) support practitioners in identifying appropriate treatment pathways and 2) guide researchers in developing and adapting interventions for use in primary care. Few interventions integrated implementation criteria in their design, and none were specifically designed for parents with exposure to ACEs or who screen positive for ACEs. Nevertheless, our findings suggest that several interventions (1, 2, 3, 4, 6, 7, 8, 11, 12, 13) may show evidence of benefit within this broad population. These interventions are diverse across other intervention characteristics relevant to implementation. The diversity across interventions may allow providers across diverse primary care settings to identify and adapt interventions most appropriate to their contexts. We aim to elucidate how clinicians can utilize implementation characteristics to determine which interventions are suitable for their specific settings and to offer a framework for making such decisions.

### 4.1 Limited integration of implementation criteria

Only one study (12) specifically cited incorporating implementation criteria as a study aim, and many studies did not report on intervention characteristics that are critical to practical application, such as session length (2, 5, 6, 7, 9, 10, 11) and training requirements (1, 2, 5, 6, 10, 11, 14). Research suggests, however, that incorporating such factors can reduce the evidence‐practice gap (Bauer & Kirchner, 2020). Increased reporting and evaluation of the intervention characteristics highlighted in this review may therefore enhance the effectiveness and implementation of interventions in real‐world primary care settings, which would align with efforts to scale evidence‐based interventions by incorporating implementation science (Dodge et al., 2024).

### 4.2 Early Evidence of benefit for r+ACE parents

Despite the lack of interventions designed for parents with exposure to ACEs, ten of the fourteen reviewed interventions (1, 2, 3, 4, 6, 7, 8, 11, 12, 13) were found to be effective for r+ACE parents on at least one outcome, with the largest effect sizes reported for parental outcomes (6). Notably, most of the interventions were studied within diverse populations (2, 3, 4, 6, 7, 12, 13), half were found to be more effective among parents with greater exposure to ACEs (2, 3, 7, 7, 11), and most reported high completion rates (1, 2, 3, 4, 6, 7, 8, 11, 12). These findings suggest potential applicability of the interventions to the diverse range of populations in primary care settings.

Variability across studies in the measurement of ACEs and the comparison of levels of ACEs exposure demands nuanced interpretation of these findings of effectiveness. The two most frequently utilized measures of exposure to ACEs differ in that the CTQ assesses only maltreatment but accounts for frequency and intensity. The ACEs questionnaire, conversely, accounts for broader interpersonal traumas but only measures presence or absence of exposure. Furthermore, the definition of r+ACE varied by study, so primary care providers must evaluate the relevance of the samples studied. For example, r+ACE may be defined in one study as having exposure to one or more ACEs (5) but in another as having exposure to four or more ACEs (13).

### 4.3 Existing intervention implementation in primary care

The two r+ACE effective interventions that were evaluated within primary care settings—Culturally Relevant Brief Interpersonal Psychotherapy (IPT‐B, 6) and Practical Resources for Effective Postpartum Parenting (PREPP, 2)—may offer insights for primary care providers considering interventions for this population. Both interventions were found effective for a subset of their targeted outcomes: PREPP for increasing infant daytime sleep and IPT‐B for decreasing maternal depressive symptoms. The studies of the two interventions varied on many factors, however, including that the rate of exposure to one or more forms of childhood maltreatment in the IPT‐B sample was nearly three times the rate in the PREPP sample.

The interventions aligned on two key factors: 1) they were offered at least in part prenatally in obstetrics clinics, and 2) they were two of the three interventions with the least number of sessions required (i.e., eight acute sessions for IPT‐B and three sessions for PREPP). These findings may suggest that the frequency of prenatal obstetrics visits may make such visits particularly suitable for intervention but that primary care implementation still requires brief treatment options. These takeaways notably align with existing research that recommends behavioral health interventions in primary care of six or fewer 30‐minute appointments (Funderburk et al., 2018).

We considered the possibility that additional interventions reviewed in this study had been implemented in primary care settings and reported on in studies outside the scope of our search. The authors, therefore, conducted brief searches for additional studies related to each reviewed intervention and found only one relevant study. LePlatte et al., 2012 tested the Mom Power (12) program among 23 teenage mothers of young children within an integrated primary care clinic specialized for teenagers. Many of the teenage mothers reported experiencing ACEs such as emotional abuse by a parent and having a family member sent to jail. Following the 10‐week program, they reported decreased depressive symptoms, PTSD symptoms, and guilt related to their parenting skills. These findings, despite clear limitations such as small sample size, suggest that a brief perinatal intervention like Mom Power may be effective even within a group with mixed ACEs history and symptoms. The study findings also suggest that full integration between medical and psychological services within the primary care setting may be required to achieve desired outcomes. The study did not report completion or utilization rates for the intervention, and primary care decision‐makers may need to evaluate whether a 10‐week program is acceptable to patients in an integrated primary care setting.

### 4.4 Diversity of interventions and diversity of primary care settings

The interventions reviewed show striking diversity across sample demographics, intervention intensity, training requirements, and other factors critical for implementation. This diversity is mirrored in the diversity of primary care settings. An analysis of the Medical Organizations Survey in the U.S. found that primary care settings vary widely by many characteristics that may influence intervention implementation (Levine et al., 2018). These characteristics include number of physicians, availability of nurse practitioners or physician assistants, ownership (e.g., independent, hospital, nonprofit, government), practice capabilities (e.g., coordination of care by a case manager), and payment orientation (e.g., percentage of patients using Medicaid). Primary care panel sizes, or number of patients seen per physician per year, range from as low as 200 in concierge practices up to and above the U.S. average of 2300 (Altschuler et al., 2012). Integration of behavioral health services also varies. Across the U.S., approximately 44% of primary care practices are collocated with behavioral health providers (Richman et al., 2020; Staab et al., 2022). Some of the remaining primary care settings offer fully integrated behavioral health programs, contrasting with others that offer no behavioral health services at all (Richman et al., 2020; Staab et al., 2022). The diversity across interventions and primary care settings suggests that there is not a single intervention or set of interventions that is optimal across primary care settings. Instead, decision‐makers in such settings must evaluate, match, and adapt existing interventions to meet their specific needs.

### 4.5 Evaluation framework for intervention‐setting fit

**FIGURE 2:**
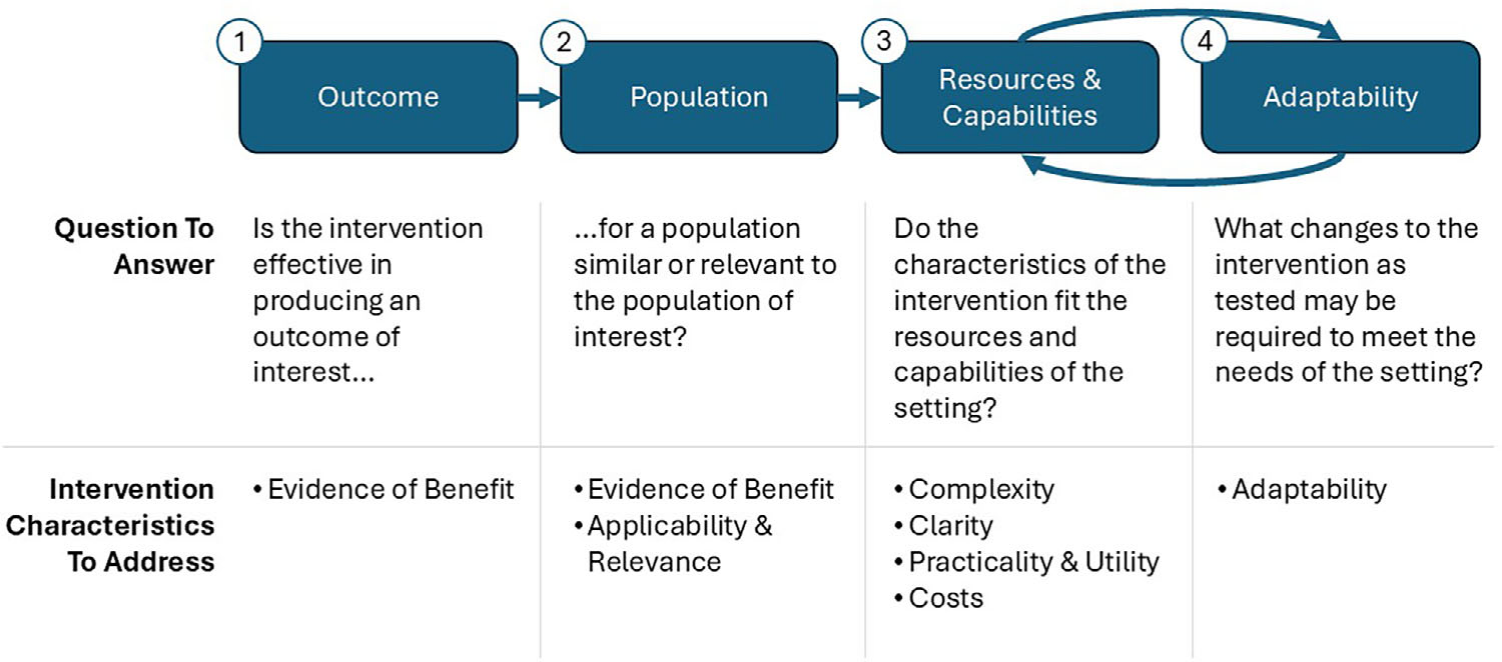
Evaluation framework for intervention‐setting fit in primary care.

The findings from the current review offer insight into how primary care decision‐makers can approach the process of evaluating the fit of a potential intervention for their patients and settings. We propose an evaluation framework with a staged process that takes into account variability in the characteristics of the interventions that are critical for implementation, as identified by Lau et al. (2015). Figure 2 outlines the proposed process.

To consider any intervention for broader implementation, it must demonstrate evidence of benefit. The first two steps of the evaluation framework pertain to this intervention characteristic identified as necessary for closing evidence‐practice gaps in primary care (Lau et al., 2015). The first step of the framework asks decision‐makers to consider if evidence supports the use of the intervention for an outcome of interest. Although the current findings highlight that most of the reviewed interventions were found effective, the diversity within ACEs suggests that the impact of ACEs cannot be addressed with a single intervention or single target outcome. Our findings highlight that the outcomes for which the reviewed interventions were found effective varied considerably, from increasing paternal amygdala reactivity toward infant crying to increasing infant daytime sleep. Primary care settings must, therefore, consider what outcomes they are equipped to address for a given parent and for parents across their practice. For example, a family medicine practice that treats both parents and their children may be better equipped to address maternal depressive or PTSD symptoms than a pediatric practice that screens for parental ACEs in hopes of improving behavioral outcomes for their pediatric patients.

The reviewed studies further reflect significant diversity in the demographics and characteristics of the samples used. The studies had fundamental differences in the comparison of r+ACE parents with those with less exposure to ACEs. Primary care decision‐makers thus must acknowledge that the evidence of benefit demonstrated in the respective studies may not generalize to all parents with exposure to ACEs. Accordingly, the second step of the evaluation framework incorporates two additional interventions characteristics identified by Lau et al. (2015) as critical for closing primary care evidence‐practice gaps: evidence of benefit and applicability and relevance. Both of these characteristics relate to whether the evidence supports the use of the intervention within the specific population of interest.

Only after decision‐makers confirm that there is evidence of benefit for the outcomes and populations of interest do we recommend that they consider the four remaining intervention characteristics: complexity, clarity, practicality and utility, and costs. These four characteristics all relate to the fit of the resources and capabilities of the setting with the demands of the intervention. This step allows primary care decision‐makers to account for the extraordinary breadth in each of these characteristics across effective interventions reviewed.

Finally, decision‐makers must consider the ways in which the intervention must be adapted to fit their settings and needs. With only two interventions tested in primary care clinics and none designed specifically for parents with exposure to ACEs, adaptations will likely be required to meet the needs of a given primary care setting, its providers, and its patients. Decision‐makers within primary care settings may view the balance of the final two steps as an iterative process through which to identify interventions suitable for its providers and patients. The following vignettes demonstrate the application of the evaluation framework.

#### 4.5.1 Vignette 1: A large metropolitan hospital

A hospital in a large metropolitan area in California, in line with the state's ACEs Aware initiative, screens all adult patients, including expectant parents, for ACEs. Screening has revealed that the percentage of their patients that report four or more ACEs is higher than the national average, with a high prevalence of exposure to childhood abuse. Practitioners in the hospital's obstetrics practice want to ensure that the standard intervention they provide for perinatal depression is suitable for mothers with exposure to ACEs.

Using the evaluation framework, the obstetrics providers first consider the interventions found effective for reducing maternal depressive symptoms in r+ACE parents. In our review these include IH‐CBT (1), CBASP (3), IPT‐B (6) and Mom Power (12).

Next, the providers situate the findings in the context of their patient population. They choose to prioritize consideration of IH‐CBT and IPT‐B due to the evidence of effectiveness in patients with clinical depression, as the hospital is planning to treat patients who screen positive and then are clinically diagnosed with depression following a clinical interview. The providers find further support for these interventions because they were similarly effective for mothers with higher exposure to childhood maltreatment as for those with lower exposure.

The providers next consider the intervention‐setting match, choosing to pilot IPT‐B for four reasons. First, it costs less to offer as it requires only about half the sessions of IH‐CBT. Second, it is more practical for the hospital because it is delivered exclusively prenatally and in clinic. This is more feasible because the hospital's patients do not necessarily stay within the hospital network for pediatric care after delivery. Third, IPT‐B may offer greater clarity, as it is reported to utilize a treatment manual.

Finally, in considering adaptations, the hospital seeks to improve the practicality and cost of the intervention by training its existing behavioral health providers, primarily master's level clinicians, to deliver the intervention.

#### 4.5.2 Vignette 2: A community family medicine clinic

A community family medicine clinic seeks to develop a consistent approach to parenting support for its primarily low‐income and non‐Hispanic white patient population. Among patients there is a high prevalence of exposure to ACEs, especially household substance use in childhood. The clinic would like to offer universal access to a preventative intervention to support effective parenting practices and wants to ensure that the intervention is effective for parents with exposure to ACEs.

Within this review, five interventions were found to be effective among r+ACE parents for parenting‐related outcomes: Minding the Baby (4), FIND (7), PFR (8), a baby‐carrier intervention (11), and GABI (13). The clinic does not consider the baby‐carrier intervention because the clinic seeks evidence of behavioral outcomes for parents, and the baby‐carrier intervention was only found to impact paternal amygdala reactivity to infant crying and not any subsequent parenting behaviors.

Next, the clinic considers whether the evidence supports the use of the intervention in populations like its own. Although PFR was found effective in a similar population demographically, the intervention compared ACEs exposure based on severity, which the clinic's ACEs measure does not account for. Furthermore, PFR was only effective among parents who had both an open case of maltreatment and a moderate to severe history of childhood physical abuse.

Among the remaining interventions, the clinic chooses to prioritize FIND for further consideration due to the intervention‐setting fit. Minding the Baby and GABI, which entail 90 and 78 sessions, respectively, each require greater resources than the clinic can access.

In the final stage of the process, the clinic considers reviewing the intervention content to ensure applicability, as the FIND intervention was tested in a primarily Hispanic sample.

### 4.6 Considerations for ACEs screening

The findings in this review highlight that for effective care, parental ACEs screening may serve only as the start of a conversation between providers and parents rather than as a standalone decision‐making instrument. As critics of routine ACEs screening have suggested (Bair‐Merritt & Zuckerman, 2016), the scores on screening tools for exposure to ACEs offer only limited information about the nature of the experiences, their impact on current symptoms and functioning, and their potential to influence infant or child outcomes. As the diversity of interventions in this review suggest, the relevance of any given intervention to exposure to a particular ACE may vary widely as well. For the growing number of primary care settings offering ACEs screening, the lack of interventions designed for parents with a history of ACEs should reflect that ACEs screening is a first rather than a final step in assessing the impact of parental exposure to ACEs on child wellbeing.

### 4.7 Future directions for perinatal intervention research

Across r+ACE effective interventions, we identified several opportunities for future research to enhance their relevance to primary care settings. Two interventions (2, 6) with eight or fewer sessions already have evidence within primary care settings, and research supports brief interventions in primary care of six or fewer sessions (Funderburk et al., 2018). These findings suggest that there is meaningful opportunity for existing interventions to be adapted and new interventions to be developed that are shorter in duration than existing interventions. Second, interventions can be developed for and adapted to the obstetric setting, as only two r+ACE effective interventions (3, 6) were designed for the prenatal period. This is particularly notable because the perinatal period is the only time when certain mechanisms of trauma transfer, such as HPA axis dysregulation, might be impacted. Third, as many interventions require master's or doctoral level clinicians, intervention delivery by lower‐cost providers like allied health professionals should be tested. Fourth, none of the r+ACE effective interventions noted the use of technology, a key lever for decreasing cost and increasing access. Mobile health interventions, for example, enhance care outcomes and engagement (Marcolino et al., 2018) and may be particularly suitable in resource‐constrained primary care settings. Finally, existing interventions have focused primarily on mothers, suggesting a missed opportunity to adapt effective interventions to fathers with exposure to ACEs.

### 4.8 Limitations

The current study has several limitations. We focus on intervention characteristics that affect primary care implementation. Broader factors such as relevant public policy and organizational characteristics are also critical for clinicians to consider but were outside the scope of this review. Additionally, the definition of ACEs definition and comparison of levels of exposure to ACEs were inconsistent in the literature, which may cloud the applicability of the studies reviewed to specific populations of parents with exposure to ACEs. Also, most studies did not differentiate between individual ACEs, despite research suggesting that outcomes may differ by childhood maltreatment type (Mandelli et al., 2015; Souch et al., 2022).

Inconsistent and missing reporting on effect sizes across studies further limited the assessment of intervention effectiveness. To be comprehensive in our findings, we included all reported outcome variables for each included study; however, each study included several outcome variables, increasing the probability of Type I error in the findings reported. Although the findings of the included studies would benefit from further replication, we believe the reported findings are relevant for decision‐makers within primary care settings currently grappling with challenges related to parental exposure to ACEs. Furthermore, the current review did not evaluate the risk for bias within the included studies. Additional validation of the findings of each study is thus required, including testing interventions specifically among parents with exposure to ACEs or parents who screen positive for ACEs.

## 5 CONCLUSION

The current study sought to evaluate existing perinatal interventions for parents with exposure to ACEs to assess their applicability to primary care settings because these settings offer high‐potential points of intervention to reach this population. Our findings suggest that several existing interventions show evidence of benefit for a range of outcomes among parents with exposure to ACEs. The diversity among these interventions on factors identified as critical to successful implementation in primary care mirrors the diversity in primary care settings. Primary care clinicians, increasingly tasked with screening for and addressing parental ACEs, can utilize the factors detailed in this study to match and adapt such interventions to enhance their practical applicability to their own patient populations.

## CONFLICT OF INTEREST STATEMENT

The authors declare no conflicts of interest.

## FUNDING INFORMATION

The authors declare no relevant funding.

## Supporting information



Supplementary material: imhj70095‐sup‐0001‐Tables.docx

## Data Availability

The authors confirm that the data supporting the findings of this study are available within the article and its supplementary materials.
