# Supplementary material for: Perinatal interventions for parents with exposure to adverse childhood experiences: A narrative review of suitability for implementation in primary care
Source: Infant Ment Health J. 2026 May 8;47:e70095. doi: 10.1002/imhj.70095 (PMC13155650; doi:10.1002/imhj.70095)
Supplement: Supplementary file 1 — Supplementary material: imhj70095‐sup‐0001‐Tables.docx [file IMHJ-47-0-s001.docx]

**Supplemental Table 1**. Characteristics of Intervention Effectiveness Detailed

|  |  |  |  |  | **Significant Main Effect of Treatment** | | | | | |
| --- | --- | --- | --- | --- | --- | --- | --- | --- | --- | --- |
|  |  |  |  |  | **Among relatively higher ACE** | | **Among relatively lower ACE** | | **Among full sample** | |
| **Study #** | **Study (first author)** | **Intervention Name** | **Outcome** | **Primary or Secondary Outcome** | **Yes / No** | **Effect Size** | **Yes / No** | **Effect Size** | **Yes / No** | **Effect Size** |
| (1) | Ammerman et al., 2016; Ammerman et al., 2013a; Ammerman et al., 2013b | In-Home Cognitive Behavioral Therapy | Maternal depressive symptoms | Primary | -- | -- | -- | -- | Yes | Least squares method: 0.55-0.90 |
|  |  |  | Maternal interpersonal support | Secondary | -- | -- | -- | -- | No | Least squares method: 0.38-0.65 (small to moderate) |
|  |  |  | Maternal social network | Secondary | -- | -- | -- | -- | No | -- |
| (2) | Berry et al., 2021 | Practical Resources for Effective Postpartum Parenting | Maternal depressive symptoms | Primary | No | -- | Yes | -- | Yes | -- |
|  |  |  | Maternal anxiety symptoms | Primary | No | -- | Yes | -- | Yes | -- |
|  |  |  | Infant daytime sleep | Secondary | Yes | -- | Yes | -- | Yes | -- |
|  |  |  | Infant night sleep | Secondary | -- | -- | -- | -- | No | -- |
|  |  |  | Infant day fuss | Secondary | -- | -- | -- | -- | No | -- |
|  |  |  | Infant night fuss | Secondary | -- | -- | -- | -- | No | -- |
|  |  |  | Infant day cry | Secondary | -- | -- | -- | -- | No | -- |
|  |  |  | Infant night cry | Secondary | -- | -- | -- | -- | No | -- |
| (3) | Blalock et al., 2013 & Cinciripini et al., 2010 | Cognitive Behavioral Analysis System of Psychotherapy | Maternal smoking abstinence | Primary | -- | -- | -- | -- | No | -- |
|  |  |  | Maternal depressive symptoms | Primary | -- | -- | -- | -- | Yes | -- |
| (4) | Condon et al., 2022 | Minding the Baby | Maternal pre-mentalizing modes | Primary | -- | -- | -- | -- | No | -- |
|  |  |  | Maternal interest/curiosity in mental states | Primary | -- | -- | -- | -- | No | -- |
|  |  |  | Maternal hostile/coercive parenting | Primary | -- | -- | -- | -- | Yes | -- |
| (5) | Goldstein et al., 2024 | Mothers and Babies Personalized | Maternal depression symptoms | Primary | No | -- | Yes | -- | -- | -- |
|  |  |  | Maternal perceived stress | Primary | No | -- | Yes | -- | -- | -- |
|  |  |  | Maternal anxiety | Primary | No | -- | No | -- | -- | -- |
|  |  |  | Maternal mood regulation | Primary | No | -- | Yes | -- | -- | -- |
|  |  |  | Maternal behavioral activation | Primary | No | -- | No | -- | -- | -- |
| (6) | Grote et al., 2012 & Grote et al., 2009 | Culturally Relevant Brief Interpersonal Psychotherapy | Maternal depression diagnosis | Primary | -- | -- | -- | -- | Yes | Cohen's *h* = 0.96-1.22 (large) |
|  |  |  | Maternal depressive symptoms | Primary | -- | -- | -- | -- | Yes | Cohen's *h* = 1.08-1.17 (large) |
|  |  |  | Maternal anxiety symptoms | Secondary | -- | -- | -- | -- | No | -- |
|  |  |  | Maternal social functioning | Secondary | -- | -- | -- | -- | No | -- |

|  |  |  |  |  | **Significant Main Effect of Treatment** | | | | | |
| --- | --- | --- | --- | --- | --- | --- | --- | --- | --- | --- |
|  |  |  |  |  | **Among relatively higher ACE** | | **Among relatively lower ACE** | | **Among full sample** | |
| **Study #** | **Study (first author)** | **Intervention Name** | **Outcome** | **Primary or Secondary Outcome** | **Yes / No** | **Effect Size** | **Yes / No** | **Effect Size** | **Yes / No** | **Effect Size** |
| (7) | Liu et al., 2021 | Filming Interactions to Nurture Development | Parental sense of competence | Primary | -- | -- | -- | -- | Yes | -- |
|  |  |  | Parental self-efficacy in nurturance | Primary | -- | -- | -- | -- | No | -- |
|  |  |  | Parental self-efficacy in discipline | Primary | -- | -- | -- | -- | No | -- |
|  |  |  | Parental self-efficacy in teaching | Primary | -- | -- | -- | -- | Yes | -- |
|  |  |  | Parental self-efficacy: instrumental care | Primary | -- | -- | -- | -- | No | -- |
|  |  |  | Child internalizing problems | Primary | -- | -- | -- | -- | Indirect effect through parental self-efficacy in teaching | -- |
|  |  |  | Child externalizing problems | Primary | -- | -- | -- | -- | No | -- |
| (8) | Pasalich et al., 2019 | Promoting First Relationships | Parental sensitivity | Primary | -- | -- | -- | -- | Yes | β = 0.25 |
|  |  |  | Child secure base behavior | Primary | Yes, for indirect effect through parental sensitivity for physical abuse | -- | No, for indirect effect through parental sensitivity for physical abuse | -- | Indirect effect through parental sensitivity | -- |
| (9) | Perrone et al., 2021 | Attachment and Biobehavioral Catch-up | Parental depression symptoms | Primary | -- | -- | -- | -- | Not significant, but trend-level finding | -- |
|  |  |  | Parental sensitivity | Primary | -- | -- | -- | -- | Yes | Cohen's *d* = 0.21 (small) |
|  |  |  | Parental intrusiveness | Primary | -- | -- | -- | -- | No | -- |
|  |  |  | Parental positive regard | Primary | -- | -- | -- | -- | No | -- |
| (10) | Ribaudo et al., 2022 | Michigan Model of Infant Mental Health-Home Visiting | Child attachment | Primary | -- | -- | -- | -- | Not significant, but trend-level finding | -- |
|  |  |  | Child initiative | Primary | -- | -- | -- | -- | No | -- |
| (11) | Riem et al., 2021 | Baby carrier intervention | Paternal salivary oxytocin | Primary | -- | -- | -- | -- | No | -- |
|  |  |  | Paternal reactivity - amygdala | Primary | -- | -- | -- | -- | Yes | -- |
|  |  |  | Paternal reactivity - insula | Primary | -- | -- | -- | -- | No | -- |
|  |  |  | Paternal reactivity - interior frontal gyrus | Primary | -- | -- | -- | -- | No | -- |
|  |  |  | Paternal reactivity - middle frontal gyrus | Primary | -- | -- | -- | -- | No | -- |
|  |  |  | Paternal reactivity - middle temporal gyrus | Primary | -- | -- | -- | -- | No | -- |
|  |  |  | Paternal reactivity - superior temporal gyrus | Primary | -- | -- | -- | -- | No | -- |
|  |  |  | Paternal reactivity - supramarginal gyrus | Primary | -- | -- | -- | -- | No | -- |
| (12) | Rosenblum et al., 2017 | Mom Power | Maternal depressive symptoms | Primary | Yes | *d* = 0.462 | -- | -- | No | -- |
|  |  |  | Maternal PTSD symptoms | Primary | Yes | *d* = 0.401 | -- | -- | Yes | -- |
|  |  |  | Maternal parenting stress | Primary | Not significant, but trend-level finding | *d* = 0.653 | -- | -- | Yes | -- |
|  |  |  | Maternal caregiving helplessness | Primary | No | *d* = 0.351 | -- | -- | -- | -- |
|  |  |  | Child caregiving behavior | Primary | No | *d* = -0.058 | -- | -- | -- | -- |

|  |  |  |  |  | **Significant Main Effect of Treatment** | | | | | |
| --- | --- | --- | --- | --- | --- | --- | --- | --- | --- | --- |
|  |  |  |  |  | **Among relatively higher ACE** | | **Among relatively lower ACE** | | **Among full sample** | |
| **Study #** | **Study (first author)** | **Intervention Name** | **Outcome** | **Primary or Secondary Outcome** | **Yes / No** | **Effect Size** | **Yes / No** | **Effect Size** | **Yes / No** | **Effect Size** |
| (13) | Steele et al., 2019 | Group Attachment-Based Intervention | Maternal supportive presence | Primary | -- | -- | -- | -- | Yes | Partial η² = 0.12 |
|  |  |  | Maternal hostility | Primary | -- | -- | -- | -- | Yes | Partial η² = 0.05 |
|  |  |  | Dyadic constriction | Primary | -- | -- | -- | -- | Yes | Partial η² = 0.16 (moderate) |
|  |  |  | Dyadic reciprocity | Primary | -- | -- | -- | -- | Yes | Partial η² = 0.19 (moderate) |
| (14) | van der Asdonk et al., 2021 | Attachment Video-feedback Intervention | Parent-child interactive quality | Primary | -- | -- | -- | -- | Yes | β = 0.26-0.35 |

Abbreviations: ACE, adverse childhood experiences.

**Supplemental Table 1**. Characteristics of Intervention Effectiveness Detailed (Continued)

| **Study #** | **Study (first author, year)** | **Intervention Name** | **Outcome** | **Intervention Effectiveness for r+ACE Parents** | **Type of Control** | **Description of Control** | **Length of Follow-up** |
| --- | --- | --- | --- | --- | --- | --- | --- |
| (1) | Ammerman et al., 2016; Ammerman et al., 2013a; Ammerman et al., 2013b | In-Home Cognitive Behavioral Therapy | Maternal depressive symptoms | Similarly effective | Passive | Standard home visiting only | 3 months |
|  |  |  | Maternal interpersonal support | Similarly effective |  |  |  |
|  |  |  | Maternal social network | More effective, for emotional abuse history only |  |  |  |
| (2) | Berry et al., 2021 | Practical Resources for Effective Postpartum Parenting | Maternal depressive symptoms | Less effective | Active, not time-matched | Treatment as usual plus two sessions with psychologist, psychoeducation, referrals, local services information | 6 weeks postpartum |
|  |  |  | Maternal anxiety symptoms | Less effective |  |  |  |
|  |  |  | Infant daytime sleep | More effective |  |  |  |
|  |  |  | Infant night sleep | -- |  |  |  |
|  |  |  | Infant day fuss | -- |  |  |  |
|  |  |  | Infant night fuss | -- |  |  |  |
|  |  |  | Infant day cry | -- |  |  |  |
|  |  |  | Infant night cry | -- |  |  |  |
| (3) | Blalock et al., 2013 & Cinciripini et al., 2010 | Cognitive Behavioral Analysis System of Psychotherapy | Maternal smoking abstinence | Less effective | Active, time-matched | Discussion of standardized topics but not solution-focused exercises | 6 months postpartum |
|  |  |  | Maternal depressive symptoms | More effective |  |  |  |
| (4) | Condon et al., 2022 | Minding the Baby | Maternal pre-mentalizing modes | -- | Passive | Treatment as usual, incl. monthly information sheets | Post-intervention |
|  |  |  | Maternal interest/curiosity in mental states | -- |  |  |  |
|  |  |  | Maternal hostile/coercive parenting | Similarly effective |  |  |  |
|  |  |  | Maternal supportive/engaged parenting | -- |  |  |  |
| (5) | Goldstein et al., 2024 | Mothers and Babies Personalized | Maternal depression symptoms | Less effective | Passive | Treatment as usual plus stress monitoring | 3 months |
|  |  |  | Maternal perceived stress | Less effective |  |  |  |
|  |  |  | Maternal anxiety | -- |  |  |  |
|  |  |  | Maternal mood regulation | -- |  |  |  |
|  |  |  | Maternal behavioral activation | -- |  |  |  |
| (6) | Grote et al., 2012 & Grote et al., 2009 | Culturally Relevant Brief Interpersonal Psychotherapy | Maternal depression diagnosis | -- | Active, not time-matched | Treatment as usual plus psychoeducation and encouragement to seek care at behavioral health center | 6 months postpartum |
|  |  |  | Maternal depressive symptoms | Similarly effective |  |  |  |
|  |  |  | Maternal anxiety symptoms | Similarly effective |  |  |  |
|  |  |  | Maternal social functioning | Similarly effective |  |  |  |
| (7) | Liu et al., 2021 | Filming Interactions to Nurture Development | Parental sense of competence | Similarly effective | Active, not time-matched | Early Head Start only | Post-intervention |
|  |  |  | Parental self-efficacy in nurturance | -- |  |  |  |
|  |  |  | Parental self-efficacy in discipline | -- |  |  |  |
|  |  |  | Parental self-efficacy in teaching | More effective |  |  |  |
|  |  |  | Parental self-efficacy: instrumental care | -- |  |  |  |
|  |  |  | Child internalizing problems | -- |  |  |  |
|  |  |  | Child externalizing problems | -- |  |  |  |
| (8) | Pasalich et al., 2019 | Promoting First Relationships | Parental sensitivity | More effective, for physical abuse history only | Active, not time-matched | Brief resource and referral service (three phone calls plus mailed information) | 6 months |
|  |  |  | Child secure base behavior | More effective, for physical abuse history only |  |  |  |

| **Study #** | **Study (first author, year)** | **Intervention Name** | **Outcome** | **Intervention Effectiveness for r+DT Parents** | **Type of Control** | **Description of Control** | **Length of Follow-up** |
| --- | --- | --- | --- | --- | --- | --- | --- |
| (9) | Perrone et al., 2021 | Attachment and Biobehavioral Catch-up | Parental depression symptoms | -- | Passive | Waitlist | Average 7.19 months |
|  |  |  | Parental sensitivity | Not significant, but trend-level finding of being less effective |  |  |  |
|  |  |  | Parental intrusiveness | -- |  |  |  |
|  |  |  | Parental positive regard | -- |  |  |  |
| (10) | Ribaudo et al., 2022 | Michigan Model of Infant Mental Health-Home Visiting | Child attachment | Less effective | (Not reported) | (Not reported) | 12 months |
|  |  |  | Child initiative | -- |  |  |  |
| (11) | Riem et al., 2021 | Baby carrier intervention | Paternal salivary oxytocin | -- | Active, time matching not reported | Received infant car seat | 1 week |
|  |  |  | Paternal reactivity - amygdala | More effective |  |  |  |
|  |  |  | Paternal reactivity - insula | -- |  |  |  |
|  |  |  | Paternal reactivity - interior frontal gyrus | -- |  |  |  |
|  |  |  | Paternal reactivity - middle frontal gyrus | -- |  |  |  |
|  |  |  | Paternal reactivity - middle temporal gyrus | -- |  |  |  |
|  |  |  | Paternal reactivity - superior temporal gyrus | -- |  |  |  |
|  |  |  | Paternal reactivity - supramarginal gyrus | -- |  |  |  |
| (12) | Rosenblum et al., 2017 | Mom Power | Maternal depressive symptoms | -- | Active, not time-matched | Two individual sessions and 10 weekly mailings of Mom Power curriculum | Post-intervention |
|  |  |  | Maternal PTSD symptoms | -- |  |  |  |
|  |  |  | Maternal parenting stress | -- |  |  |  |
|  |  |  | Maternal caregiving helplessness | -- |  |  |  |
|  |  |  | Child caregiving behavior | -- |  |  |  |
| (13) | Steele et al., 2019 | Group Attachment-Based Intervention | Maternal supportive presence | Similarly effective | Active, not time-matched | Systematic Training for Effective Parenting (STEP), 10- to 12-week weekly intervention | Post-intervention |
|  |  |  | Maternal hostility | Similarly effective |  |  |  |
|  |  |  | Dyadic constriction | Less effective |  |  |  |
|  |  |  | Dyadic reciprocity | Not significant, but trend-level finding of being less effective |  |  |  |
| (14) | van der Asdonk et al., 2021 | Attachment Video-feedback Intervention | Parent-child interactive quality | Less effective | (1) Passive and (2) Active, time matching not reported | (1) Standard CPS parenting capacity assessment (2) Psychoeducational intervention | Approximately 2 months |

Abbreviations: r+ACE, relatively higher exposure to adverse childhood experiences; CPS, child protective services.

*Note.* Interventions were considered similarly or more effective for r+ACE parents if outcomes met either of two criteria: (1) there was a significant main effect of treatment versus control among r+ACE parents, OR (2) there were both: (a) a significant main effect of treatment versus control for a full sample that included r+ACE parents and relatively lower ACE (r-ACE) parents AND (b) either (i) no significant interaction effect of treatment and relative ACE exposure status (and time, where relevant), indicating similar effectiveness across relative ACE exposure status or (ii) a significant interaction effect in the direction of greater effectiveness for r+ACE parents.

**Supplemental Table 2**. Characteristics of Intervention Implementation Detailed

| **Study #** | **Study (first author, year)** | **Intervention** | **Location** | **ACEs Exposure Assessment** | **Designed for ACE History?** | **Program Timing** | **Completion Rates** | **Completion Definition** | **Other Utilization / Acceptability** |
| --- | --- | --- | --- | --- | --- | --- | --- | --- | --- |
| (1) | Ammerman et al., 2016; Ammerman et al., 2013a; Ammerman et al., 2013b | In-Home Cognitive Behavioral Therapy (IH-CBT) | USA | CTQ-SF | No | Pregnancy through postpartum | 86% | Completed all assessment points | (Not reported) |
| (2) | Berry et al., 2021 | Practical Resources for Effective Postpartum Parenting (PREPP) | USA | CTQ-SF | No | Pregnancy through postpartum | 83-100% | Completed all sessions | (Not reported) |
| (3) | Blalock et al., 2013 & Cinciripini et al., 2010 | Cognitive Behavioral Analysis System of Psychotherapy (CBASP) | USA | CTQ-SF | No | Pregnancy | 78% | Completed at least 7 of 10 sessions | 74% completed 8 sessions, 70% completed 9 sessions |
| (4) | Condon et al., 2022 | Minding the Baby (MtB) | USA | CTQ-SF | No | Pregnancy through postpartum | 71-83% | Completed program, but may have missed sessions | (Not reported) |
| (5) | Goldstein et al., 2024 | Mothers and Babies Personalized (MB-P) | USA | ACEs Questionnaire - modified | No | Pregnancy | Not reported | Not reported | (Not reported) |
| (6) | Grote et al., 2012 & Grote et al., 2009 | Culturally Relevant Brief Interpersonal Psychotherapy (IPT-B) | USA | CTQ-SF | No | Pregnancy | 68% | Completed at least 7 sessions | (Not reported) |
| (7) | Liu et al., 2021 | Filming Interactions to Nurture Development (FIND) | USA | Unvalidated ACEs measure | No | Postpartum | 65.9% | Completed all research visits | Mean dosage 4.69 of 5 sessions; 76.9% of families completed sessions in all five core elements |
| (8) | Pasalich et al., 2019 | Promoting First Relationships (PFR) | USA | CTQ-SF | No | Postpartum | 86% | Completed all 10 sessions | (% participants, # sessions completed): (15%, 0); (7% 1); (4%, 2); (1%, 3); (5%, 4); (4%, 7); (1%, 8) |
| (9) | Perrone et al., 2021 | Attachment and Biobehavioral Catch-up (ABC) | USA | ACEs Questionnaire | No | Postpartum | 31.5% | Completed 10 sessions | (Not reported) |
| (10) | Ribaudo et al., 2022 | Michigan Model of Infant Mental Health-Home Visiting (IMH-HV) | USA | ACEs Questionnaire | No | Postpartum | Not reported | Not reported | (Not reported) |
| (11) | Riem et al., 2021 | Baby carrier intervention (BCI) | The Netherlands | CTS | No | Postpartum | 92.3% | Received instructional intervention | Mean carrier use = 11.72 hours over three weeks; instructued to use for 18 hours over three weeks |

| **Study #** | **Study (first author, year)** | **Intervention** | **Location** | **ACEs Exposure Assessment** | **Designed for ACE History?** | **Program Timing** | **Completion Rates** | **Completion Definition** | **Other Utilization / Acceptability** |
| --- | --- | --- | --- | --- | --- | --- | --- | --- | --- |
| (12) | Rosenblum et al., 2017 | Mom Power (MP) | USA | LSC - modified | Designed for mothers with any trauma or abuse history | Postpartum | 66% | Completed at least 7 of 10 group sessions | Average 6 group sessions received; 96% completers felt better able to understand child needs; 92% completers felt supported from other mothers in group |
| (13) | Steele et al., 2019 | Group Attachment-Based Intervention (GABI) | USA | Clinical ACEs Questionnaire | Designed for mothers with trauma history or other risk factors for child maltreatment | Postpartum | 40.4% | Attended at least "a few" sessions | (Not reported) |
| (14) | van der Asdonk et al., 2021 | Attachment Video-feedback Intervention (AVI) | Canada | CTQ | No | Postpartum | 71.5% | Completed post-intervention visits | (Not reported) |

Abbreviations: ACE, adverse childhood experience; r+ACE, relatively higher exposure to ACEs; CTQ-SF, Childhood Trauma Questionnaire – Short Form; LSC, Life Stressor Checklist; CTS, Conflict Tactics Scale – Parent Child; CTQ, Childhood Trauma Questionnaire.

**Supplemental Table 2**. Characteristics of Intervention Implementation Detailed (Continued)

| **Study #** | **Study (first author, year)** | **Intervention** | **Participants** | **Sample** | **Intervention Adaptability** | **Exposure to ACEs Prevalence** | **Sample Demographics** |
| --- | --- | --- | --- | --- | --- | --- | --- |
| (1) | Ammerman et al., 2016; Ammerman et al., 2013a; Ammerman et al., 2013b | In-Home Cognitive Behavioral Therapy (IH-CBT) | 93 mothers | Clinical: MDD diagnosis | Population and individual | >=80.4% | - 62.4% white - 92.5% non-Latina - 76.3% income <$20k |
| (2) | Berry et al., 2021 | Practical Resources for Effective Postpartum Parenting (PREPP) | 109 mother-child dyads | Community | Individual | 32.1% | - 74.5% Hispanic - 51.4% income <$25k |
| (3) | Blalock et al., 2013 & Cinciripini et al., 2010 | Cognitive Behavioral Analysis System of Psychotherapy (CBASP) | 248 pregnant women | Clinical: Tobacco use | Not reported | 76% | - 33% white, non-Hispanic - 50% income <$20k |
| (4) | Condon et al., 2022 | Minding the Baby (MtB) | 97 mother-child dyads | Community | Individual | 64% with at least one CM subtype endorsed | - 33% Black - 62% Hispanic - 91% receiving public assistance |
| (5) | Goldstein et al., 2024 | Mothers and Babies Personalized (MB-P) | 95 pregnant women | Community | Individual | 71% with at least one ACE | - 69.5% white - Mean income-to-need ratio of 5.6 |
| (6) | Grote et al., 2012 & Grote et al., 2009 | Culturally Relevant Brief Interpersonal Psychotherapy (IPT-B) | 53 pregnant women | Clinical: Depression | Population | 92% | - 63.5% Black - 84.6% income <$20k |
| (7) | Liu et al., 2021 | Filming Interactions to Nurture Development (FIND) | 91 caregiver-child dyads; 98.9% mothers | Community | Not reported | (Not reported) | - 80.2% Hispanic - 61.8% income below federal poverty level |
| (8) | Pasalich et al., 2019 | Promoting First Relationships (PFR) | 247 parent-child dyads; >90% mothers | Other: Open case of maltreatment | Not reported | 33% (moderate to severe) | - 77% white - 19% Hispanic - 79% receiving food stamps |
| (9) | Perrone et al., 2021 | Attachment and Biobehavioral Catch-up (ABC) | 200 caregiver-child dyads; 96% mothers | Community | Not reported | 51.3% with at least 4 ACEs | - 64.5% African American - 15.0% Hispanic/Latin - 83% income below poverty level |
| (10) | Ribaudo et al., 2022 | Michigan Model of Infant Mental Health-Home Visiting (IMH-HV) | 58 mother-child dyads | Community: High risk | Individual | Not reported; average ACE score 3.64 | - 62.1% racial minority - 15.8% income <$20k |
| (11) | Riem et al., 2021 | Baby carrier intervention (BCI) | 63 first-time fathers | Community | Not reported | Reported qualitatively as low | Not reported |
| (12) | Rosenblum et al., 2017 | Mom Power (MP) | 122 mother-child dyads | Community: High risk | Individual | 61% (includes interpersonal trauma from childhood and adulthood) | - 71% racial/ethnic minority - 62% income <$15k |
| (13) | Steele et al., 2019 | Group Attachment-Based Intervention (GABI) | 78 mother-child dyads; paternal engagement not reported | Community: High risk | Individual | 75.6% with at least 4 ACEs | - >90% Black, Hispanic or biracial - 44.9% unstable housing |
| (14) | van der Asdonk et al., 2021 | Attachment Video-feedback Intervention (AVI) | 88 caregiver-child dyads; 86% mothers | Other: Substantiated child maltreatment | Not reported | 87% | - 30% ethnic minority - 86% unemployed or living on social welfare |

Abbreviations: ACE, adverse childhood experience; MDD, major depressive disorder; ob/gyn, obstetrics/gynecology; CM, childhood maltreatment.

**Supplemental Table 2**. Characteristics of Intervention Implementation Detailed (Continued)

| **Study #** | **Study (first author, year)** | **Intervention** | **Treatment Standardization** | **Intervention Delivered by** | **Intervention Training Required** | **Number of Intervention Sessions** | **Intervention Session Length** | **Total Intervention Hours** | **Intervention Mode and Setting** |
| --- | --- | --- | --- | --- | --- | --- | --- | --- | --- |
| (1) | Ammerman et al., 2016; Ammerman et al., 2013a; Ammerman et al., 2013b | In-Home Cognitive Behavioral Therapy (IH-CBT) | Not reported | Master's | Not reported | 15 weekly sessions | 60 minutes | 15 hours | - Individual - In-person - Home |
| (2) | Berry et al., 2021 | Practical Resources for Effective Postpartum Parenting (PREPP) | Standardized protocol | PhD | Not reported | 3 sessions + 1 telephone call | Not reported | Not reported | - Individual - In-person - Clinic: ob/gyn |
| (3) | Blalock et al., 2013 & Cinciripini et al., 2010 | Cognitive Behavioral Analysis System of Psychotherapy (CBASP) | Treatment manual | PhD | - Must pass competency - Ongoing fidelity checks | 10 weekly sessions | 60 minutes | 10 hours | - Individual - In-person - Setting not reported |
| (4) | Condon et al., 2022 | Minding the Baby (MtB) | Treatment manual | RN and Master's | - 2-3 day intensive training - Ongoing supervision and fidelity checks | ~ 90 sessions: Weekly to 1st birthday then biweekly to 2nd birthday | ~1 hour | ~90 hours | - Individual - In-person - Home |
| (5) | Goldstein et al., 2024 | Mothers and Babies Personalized (MB-P) | Treatment manual | Not reported | Not reported | 12 sessions | Not reported | Not reported | - Individual - In-person or virtual - Setting not reported |
| (6) | Grote et al., 2012 & Grote et al., 2009 | Culturally Relevant Brief Interpersonal Psychotherapy (IPT-B) | Treatment manual | Master's and PhD | Not reported | 8 acute sessions + maintenance sessions | Not reported | Not reported | - Individual - In-person - Clinic: ob/gyn |
| (7) | Liu et al., 2021 | Filming Interactions to Nurture Development (FIND) | Activities per session coded | Master's and unspecified | - 2-3 day intensive training - Ongoing supervision and fidelity checks | 10 weekly sessions | Not reported | Not reported | - Individual - In-person - Home |
| (8) | Pasalich et al., 2019 | Promoting First Relationships (PFR) | Weekly worksheets | Master's | - ~77 hours of training over 5-6 months - Ongoing supervision and fidelity checks | 10 weekly sessions | 60 to 75 minutes | 10-12.5 hours | - Individual - In-person - Home |
| (9) | Perrone et al., 2021 | Attachment and Biobehavioral Catch-up (ABC) | Treatment manual | No minimum education requirement | - 2-3 day intensive training - Ongoing supervision and fidelity checks | 10 weekly sessions | Not reported | Not reported | - Individual - In-person - Home |
| (10) | Ribaudo et al., 2022 | Michigan Model of Infant Mental Health-Home Visiting (IMH-HV) | Not reported | Not reported | Not reported | ~50-100 sessions: 1-2x weekly visits for 12 months | Not reported | Not reported | - Individual - In-person - Home |
| (11) | Riem et al., 2021 | Baby carrier intervention (BCI) | Not reported | Not reported | Not reported | One home visit | Not reported | Not reported | - Indvididual - In-person - Home |
| (12) | Rosenblum et al., 2017 | Mom Power (MP) | Treatment manual | Master's and unspecified | - 2-3 day intensive training - Ongoing supervision and fidelity checks | 13 sessions: 3 individual + 10 group | 3 hours for group sessions | 30+ hours | - Individual and group - In-person - Home and community |
| (13) | Steele et al., 2019 | Group Attachment-Based Intervention (GABI) | Treatment manual | Not reported | - 2-3 day intensive training - Ongoing supervision and fidelity checks | ~78 sessions: 3x weekly sessions for 26 weeks | 120 minutes | Up to 156 hours | - Group - In-person - Clinic |
| (14) | van der Asdonk et al., 2021 | Attachment Video-feedback Intervention (AVI) | Treatment protocol | Bachelor's | Not reported | Maximum 12 sessions | 3 hours | Maximum 36 hours | - Individual - In-person - Setting not reported |
